# Supplementary material for: Distinct organization of two cortico-cortical feedback pathways
Source: Nat Commun. 2022 Oct 27;13:6389. doi: 10.1038/s41467-022-33883-9 (PMC9613627; doi:10.1038/s41467-022-33883-9)
Supplement: Supplementary file 1 — Supplementary Information [file 41467_2022_33883_MOESM1_ESM.pdf]

Supplementary Materials

Virus infection covered both superficial and deep layers in the injection sites

For each injection into LM or vM1, we injected the virus into two depths, 300 µm and 700 µm relative to the brain surface. The infection covered cells in both superficial and deep layers (Fig S1). In this study, we did not discriminate between the feedback projections originating from superficial or deep layers.

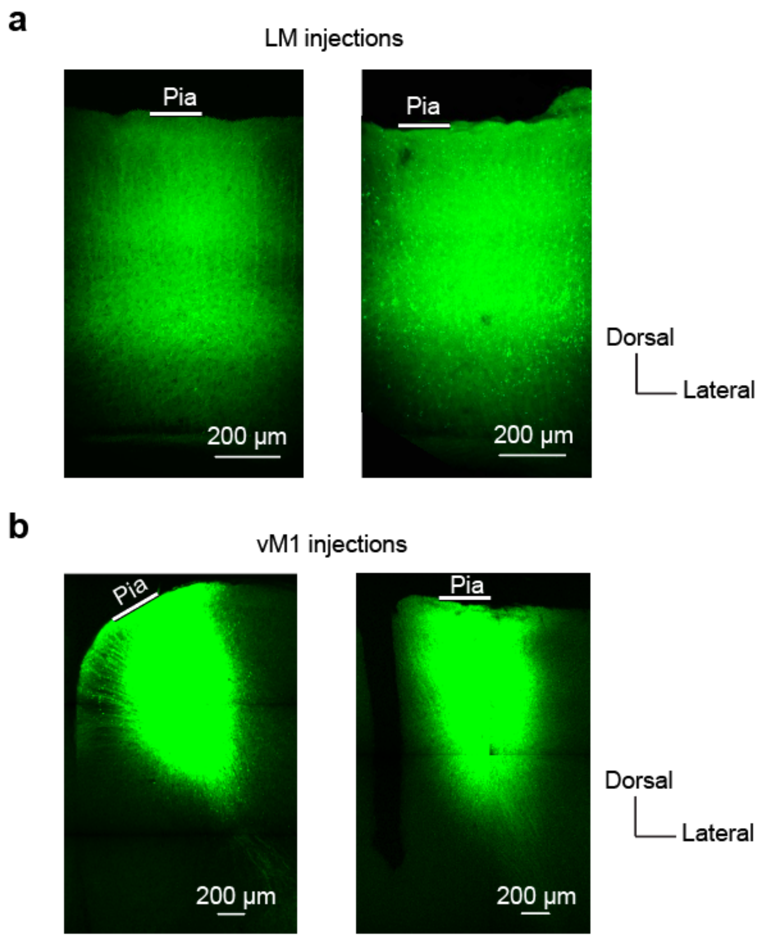

Fig. S1. Example images for virus infection at the injection sites in LM (a, 2 animals) and vM1 (b, 2 animals). Each of these images are from a different animal. Source data are provided as a Source Data file.

Confirmation of cell classes with their recovered morphologies and firing patterns

In a subset of experiments, we recovered the morphologies of the recorded cells to confirm their cell classes. Morphologies and firing patterns were particularly important in two cases in our experiments. First, it has been reported in previous studies [1–3] that a subset of SOM+ cells are fast-spiking (FS) basket cells (Fig S2a and b, bottom), while the rest of the cells are non-fast-spiking (Non-FS) cells (Fig S2a and b, top). As the two subsets of cells are very different in the morphology and firing pattern (Fig S2a and b), we excluded the activities of FS cells in the main analyses, although their log2 normalized EPSPs are not significantly different (Fig S2c and d). Second, in the bursting experiments (Fig 5), we mainly used the firing pattern to identify the intrinsic bursty cells in L5 in V1 or vS1. The firing pattern of the bursty cells was featured with their high firing rate (> 100 Hz, Fig S3) at the beginning of the current injection and the large afterhyperpolarization (AHP) following the bursting. We morphologically recovered a subset of the cells with bursty firing patterns and consistent with previous literature, these cells were thick-tufted cells (Fig S3).

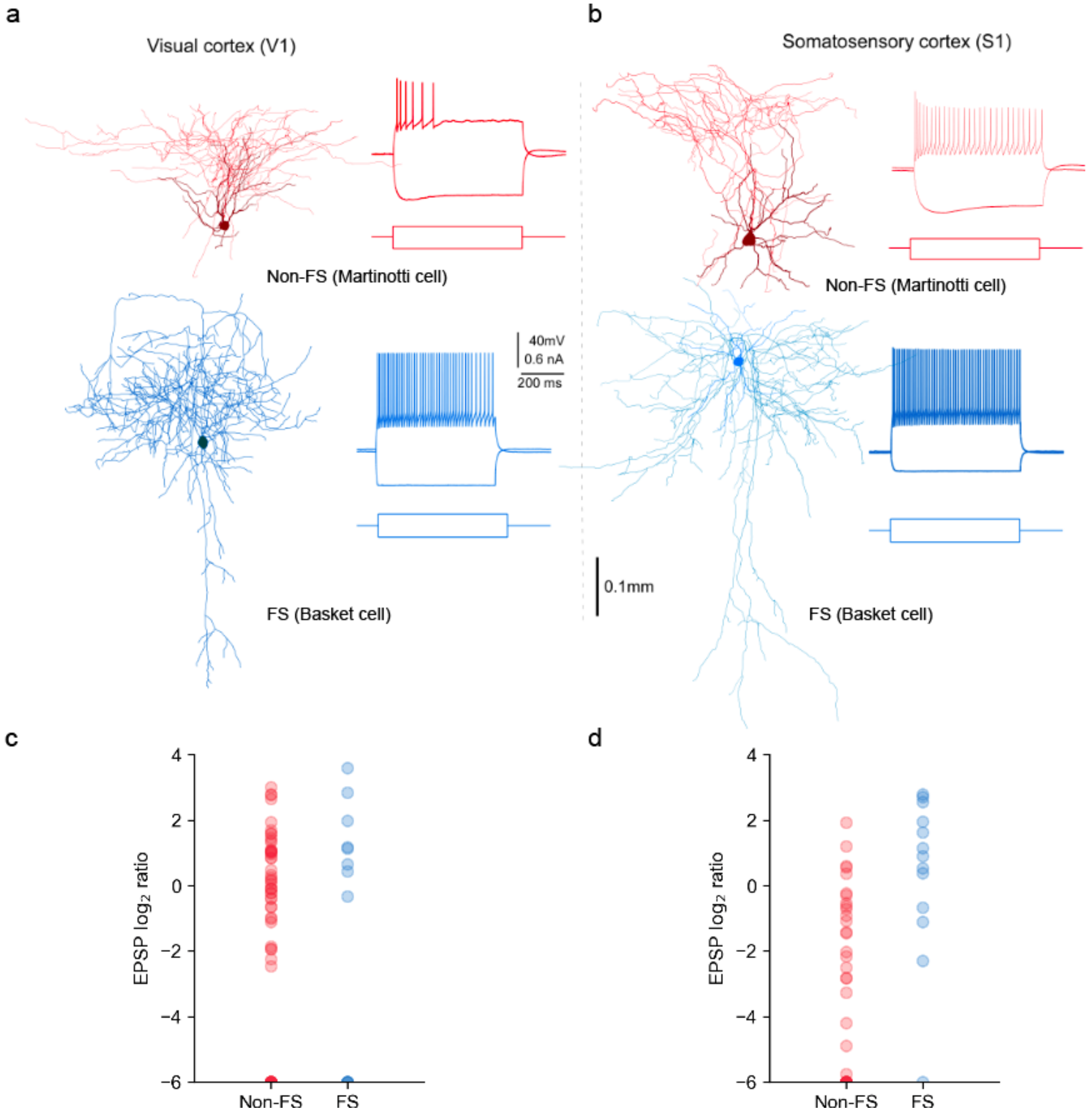

Fig S2. SOM+ cells are composed of cells with different firing patterns and morphologies. **a** Non-fast-spiking (Non-FS) cells and fast-spiking (FS) cells of SOM+ cells in V1. Example morphologies and firing patterns of Non-FS cells (top, red) and FS cells (bottom, blue). In this example, the Non-FS cell was a Martinotti cell and the FS cell was a basket cell. **b** FS cells and Non-FS cells of SOM+ cells in vS1. Example morphologies and firing patterns of Non-FS cells (top, red) and FS cells (bottom, blue). In this example, the Non-FS cell was a Martinotti cell and the FS cell was a Basket cell. **c** Log2 normalized EPSP of Non-FS cells and FS cells in V1 relative to L2/3 pyramidal cells. **d** Log2 normalized EPSP of Non-FS cells and FS cells in vS1 relative to L2/3 pyramidal cells. Source data are provided as a Source Data file.

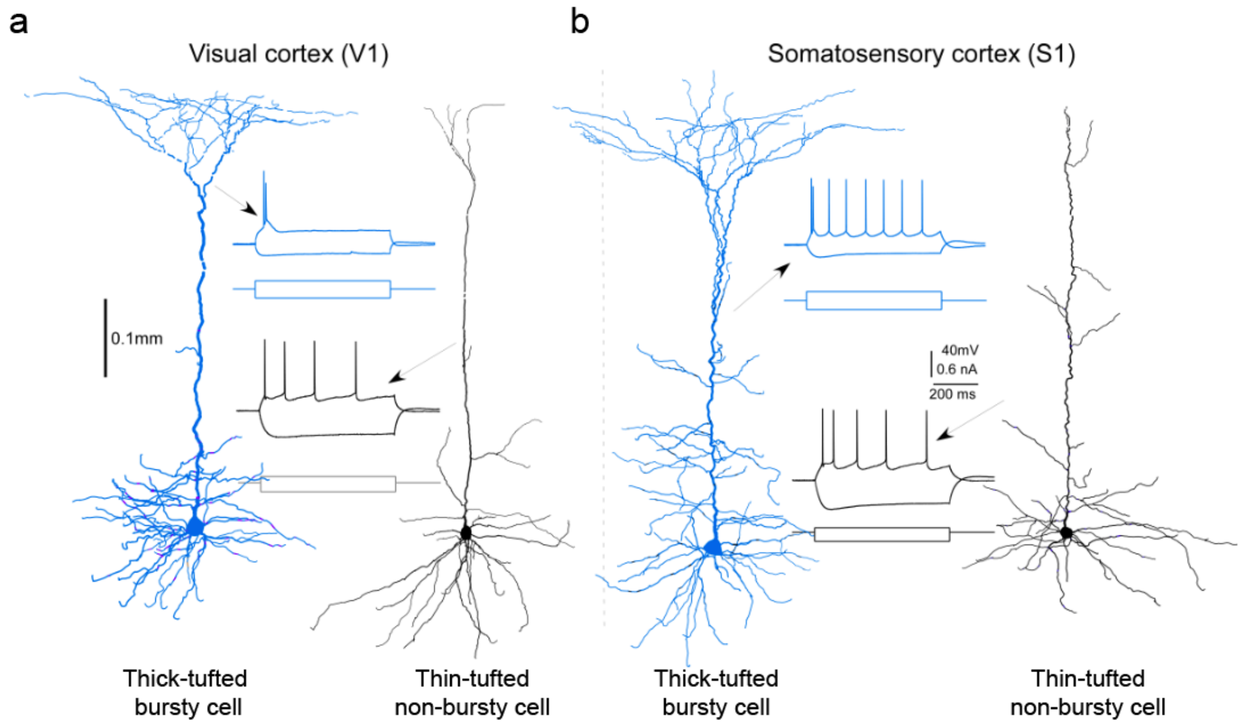

Fig S3. Intrinsic bursty cells and Non-bursty cells in L5 were identified with both the morphologies and the firing patterns. **a** Morphologies and firing patterns of example thick-tufted bursty cell (left, blue) and thin-tufted non-bursty cell (right, black) in V1. **b** Morphologies and firing patterns of example thick-tufted bursty cell (left, blue) and thin-tufted non-bursty cell (right, black) in vS1. Source data are provided as a Source Data file.

Blue LED stimulation do not elicit activities on cells without ChR2

To exclude the possibility that blue LED stimulation itself elicits electrical responses, we recorded ESPCs four cells in the visual cortex in absence of ChR2 when stimulated with 2 ms LED pulses. We found no ESPCs in these cells in response to the LED simulation (Fig S4).

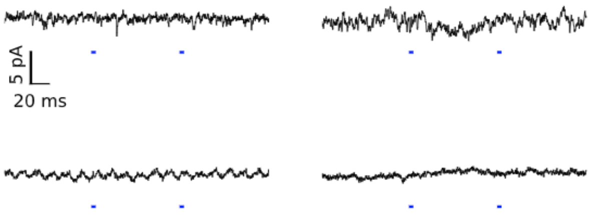

Fig S4. EPSC recording on 4 cells without ChR2 stimulated with blue LED. Cells in the visual cortex from an animal without virus injection were clamped to -70 mV and stimulated with 2 ms LED light. Source data are provided as a Source Data file.

Most of the recorded EPSC and EPSP events were monosynaptic

To identify whether the elicited events were monosynaptic, in a subset of the connectivity experiments, we applied the sodium channel blocker tetrodotoxin (TTX, 1 μM) and the potassium channel blocker 4-aminopyridine (4-AP, 500 μM) [4]. TTX blocks action potentials to prevent polysynaptic events, while 4-AP enhances ChR2-mediated depolarization of axonal boutons, increasing the detectability of monosynaptic events [4,5]. We found that all light-evoked synaptic events were silenced by TTX alone, but most of them could be partially recovered by the addition of 4-AP (Fig S5a and b), indicating that most of the excitatory responses we recorded were indeed monosynaptic. We selected 7 ms as a latency cutoff for responsive cells in all subsequent experiments, because we found that all cells that were recoverable by the addition of 4-AP had latencies less than 7 ms (Fig S5c). Although there are cells with latencies smaller than 7 ms that are not recoverable (5/43, Fig S5c), we decided not to use a shorter latency as the cutoff because of the following reasons. First, the TTX-4-AP approach is a stringent test for monosynaptic events. That is, it is almost impossible for polysynaptic responses to recover, while some small monosynaptic events may fail to recover because the presence of TTX-4-AP typically causes a significant reduction in the EPSC amplitudes [4,5]. Second, the latencies in our study are longer than typical latencies in other literature [6]. This is because our recordings were performed under room temperature instead of physiological temperature, which preserves the quality of tissues [7,8]. It is possible that some events by this standard were polysynaptic, but we found that the recorded excitatory cells were never driven to fire by feedback activation (Fig 3e), indicating a low rate of polysynaptic events. We have also used 4 ms as the latency cutoff and redid the analysis (Fig S6) and our main conclusions preserved.

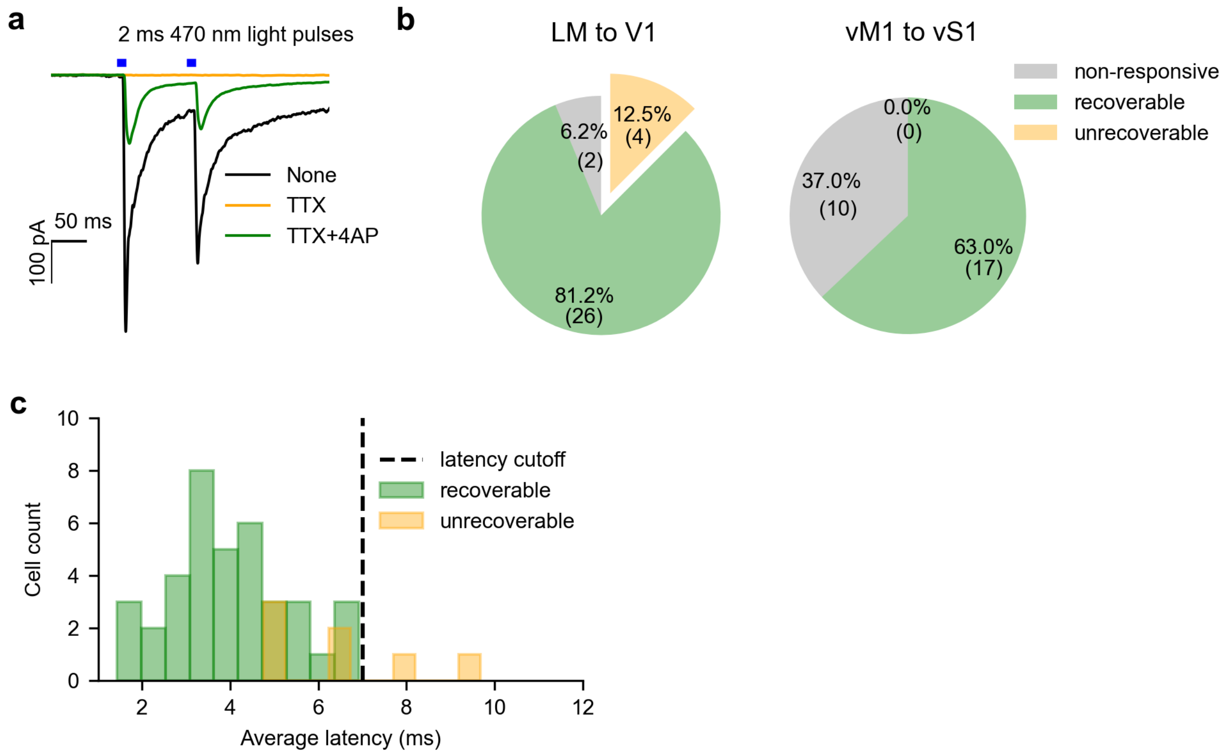

Fig S5. Identify monosynaptic events with pharmacological methods. **a** Example traces of feedback (blue ticks) responses with no drug (black), TTX (orange), and both TTX and 4-AP (green). The cells that were still responsive in the presence of TTX and 4-AP were identified as recoverable cells. **b** Proportion of cells that are non-responsive without drug (gray), recoverable (green) and unrecoverable (orange) in V1 (left) or vS1 (right). **c** Distribution of the average latencies across traces for recoverable cells and unrecoverable cells. We set 7 ms as the latency cutoff for presumably monosynaptic events. Source data are provided as a Source Data file.

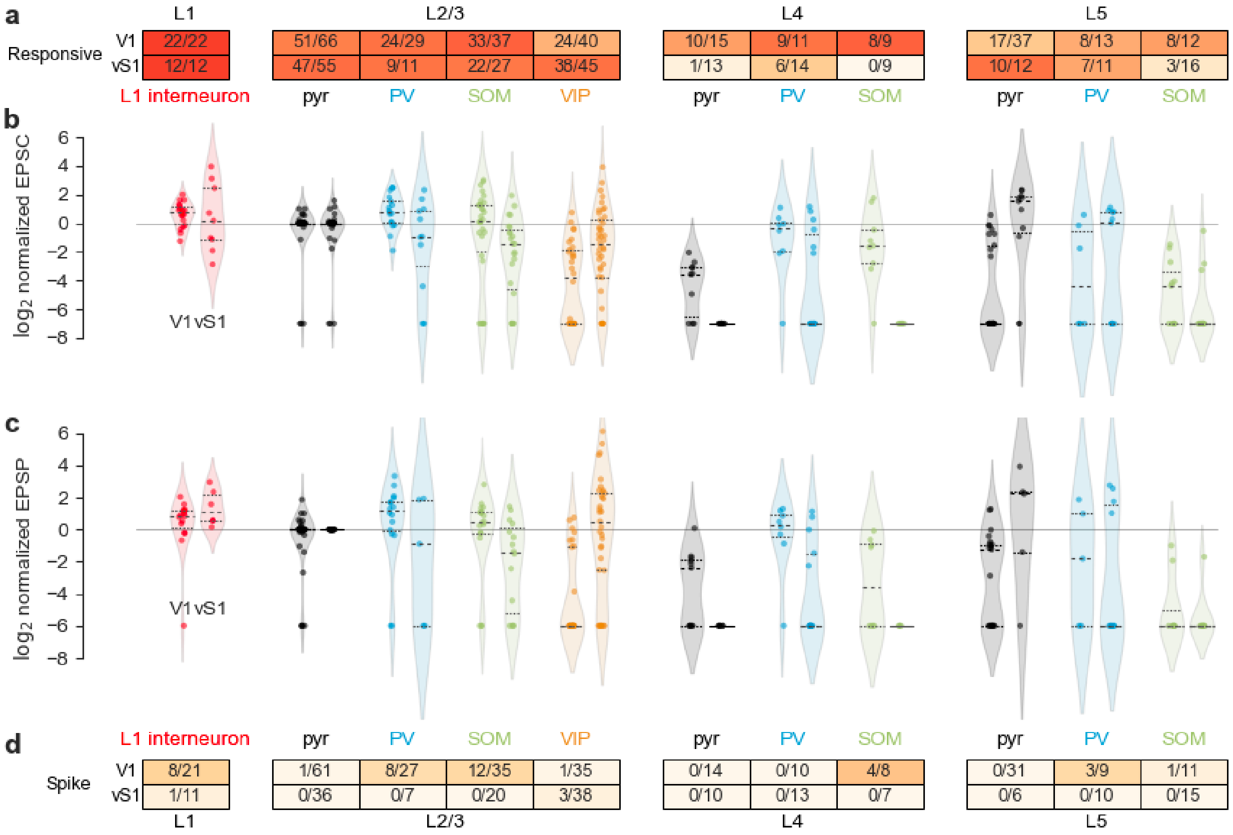

Fig S6. Connectivity results reanalyzed with 4 ms as the latency cutoff of presumably monosynaptic events, corresponding to Fig 3. **a** The proportion of responsive cells in V1 or vS1 in different layers (number of responsive cells/total number of recorded cells). The color in the table indicates the probability level, same for panel d. **b** The log2 normalized EPSC of cells in either V1 (left) or vS1 (right) normalized to the average EPSC of L2/3 pyramidal cells. Colors and positions of the violin plot indicate the cell type corresponding to the charts in panels a and d. Each dot indicates one recorded cell and the color indicates the cell type. The black dashed lines indicate the quartiles (top and bottom) and the median (middle). The outlines indicate the distributions of the log2 normalized EPSCs. **c** Same as b, for log2 normalized EPSP. **d** The proportion of spiking cells in V1 or vS1 in different layers (number of spiking cells/total cells recorded in the current-clamp mode). Source data are provided as a Source Data file.

	L1	L2/3 Exc	L2/3 PV	L2/3 SOM	L2/3 VIP	L4 Pyr	L4 PV	L4 SOM	L5 Pyr	L5 PV	L5 SOM
L1	N/A	0.0034	0.63	0.29	6.1E-09	1.5E-06	0.27	0.017	5.1E-07	0.044	0.0051
L2/3 Pyr	--	N/A	0.0056	0.76	0.0034	0.0034	0.53	0.072	0.0034	0.28	0.050
L2/3 PV	--	--	N/A	0.093	1.7E-10	1.4E-07	0.12	0.0048	2.0E-08	0.015	0.0011
L2/3	--	--	--	N/A	6.1E-09	7.1E-06	0.70	0.078	1.8E-06	0.16	0.031

SOM											
L2/3 VIP	--	--	--	--	N/A	0.66	7.7E-04	0.045	0.43	0.047	0.041
L4 Exc	--	--	--	--	--	N/A	0.0019	0.046	0.33	0.046	0.047
L4 PV	--	--	--	--	--	--	N/A	0.30	0.0054	0.40	0.20
L4 SOM	--	--	--	--	--	--	--	N/A	0.16	0.89	0.89
L5 Pyr	--	--	--	--	--	--	--	--	N/A	0.15	0.17
L5 PV	--	--	--	--	--	--	--	--	--	N/A	0.78
L5 SOM	--	--	--	--	--	--	--	--	--	--	N/A

Table S1. *p* values of LM feedback elicited log2 normalized EPSC comparison across cell classes in V1. Two-sided Conover's test for comparisons among cell classes other than the L2/3 pyramidal cells. Two-sided permutation signed test for comparisons between L2/3 pyramidal cells and other cell classes. P values were adjusted with Benjamin-Hochberg methods for multiple comparisons.

	L1	L2/3 Pyr	L2/3 PV	L2/3 SOM	L2/3 VIP	L4 Exc	L4PV	L4 SOM	L5 Pyr	L5 PV	L5 SOM
L1	N/A	0.0034	0.70	0.78	0.0043	8.9E-05	0.34	0.16	4.6E-04	0.14	0.08
L2/3 Pyr	--	N/A	0.10	0.22	0.0056	0.0056	0.68	0.51	0.0034	0.38	0.51
L2/3 PV	--	--	N/A	0.44	3.3E-04	5.4E-06	0.18	0.064	1.4E-05	0.063	0.027
L2/3 SOM	--	--	--	N/A	0.0024	3.6E-05	0.42	0.19	1.5E-04	0.17	0.094
L2/3 VIP	--	--	--	--	N/A	0.051	0.26	0.536	0.316	0.726	0.65
L4 Exc	--	--	--	--	--	N/A	0.015	0.047	0.28	0.108	0.054
L4 PV	--	--	--	--	--	--	N/A	0.69	0.073	0.603	0.563
L4 SOM	--	--	--	--	--	--	--	N/A	0.21	0.88	0.88
L5 Pyr	--	--	--	--	--	--	--	--	N/A	0.35	0.25
L5 PV	--	--	--	--	--	--	--	--	--	N/A	1.0
L5 SOM	--	--	--	--	--	--	--	--	--	--	N/A

Table S2. *p* values of LM feedback elicited log2 normalized EPSP comparison across cell classes in V1. Two-sided Conover's test for comparisons among cell classes other than the L2/3 pyramidal cells.Two-sided permutation signed test for comparisons between L2/3 pyramidal cells and other cell classes. P values were adjusted with Benjamin-Hochberg methods for multiple comparisons.

	L1	L2/3 Pyr	L2/3 PV	L2/3	L2/3 VIP	L4 Exc	L4PV	L4 SOM	L5 Pyr	L5 PV	L5 SOM

				SOM							
**L1**	N/A	0.27	0.51	0.0034	0.061	2.3E-07	0.0015	4.4E-07	0.56	0.058	3.9E-07
**L2/3 Pyr**	--	N/A	0.45	0.0034	0.0034	0.0034	0.0034	0.0056	0.021	0.09	0.0034
**L2/3 PV**	--	--	N/A	0.034	0.32	4.6E-06	0.013	7.2E-06	0.21	0.24	8.9E-06
**L2/3 SOM**	--	--	--	N/A	0.092	7.7E-04	0.49	0.0011	3.3E-04	0.51	0.0022
**L2/3 VIP**	--	--	--	--	N/A	2.4E-06	0.033	5.5E-06	0.0082	0.62	2.4E-06
**L4 Exc**	--	--	--	--	--	N/A	0.013	1	1.6E-08	6.3E-04	0.5
**L4 PV**	--	--	--	--	--	--	N/A	0.016	1.5E-04	0.24	0.043
**L4 SOM**	--	--	--	--	--	--	--	N/A	2.5E-08	8.4E-04	0.51
**L5 Pyr**	--	--	--	--	--	--	--	--	N/A	0.012	1.7E-08
**L5 PV**	--	--	--	--	--	--	--	--	--	N/A	0.0019
**L5 SOM**	--	--	--	--	--	--	--	--	--	--	N/A

Table S3. *p* values of vM1 feedback elicited log2 normalized EPSC comparison across cell classes in vS1. Two-sided Conover's test for comparisons among cell classes other than the L2/3 pyramidal cells. Two-sided permutation signed test for comparisons between L2/3 pyramidal cells and other cell classes. P values were adjusted with Benjamin-Hochberg methods for multiple comparisons.

	L1	L2/3 Pyr	L2/3 PV	L2/3 SOM	L2/3 VIP	L4 Exc	L4PV	L4 SOM	L5 Pyr	L5 PV	L5 SOM
**L1**	N/A	0.034	0.21	0.031	0.65	8.9E-05	0.0057	1.6E-04	0.76	0.25	2.3E-04
**L2/3 Pyr**	--	N/A	0.3	0.023	0.061	0.015	0.01	0.034	0.17	0.33	0.0056
**L2/3 PV**	--	--	N/A	0.49	0.23	0.0046	0.18	0.006	0.34	0.84	0.01
**L2/3 SOM**	--	--	--	N/A	0.0066	0.0092	0.43	0.014	0.073	0.29	0.023
**L2/3 VIP**	--	--	--	--	N/A	2.4E-06	5.6E-04	7.4E-06	0.94	0.28	5.5E-06
**L4 Exc**	--	--	--	--	--	N/A	0.061	1	3.2E-04	0.0011	0.72
**L4 PV**	--	--	--	--	--	--	N/A	0.075	0.017	0.075	0.13
**L4 SOM**	--	--	--	--	--	--	--	N/A	4.9E-04	0.0019	0.72
**L5 Pyr**	--	--	--	--	--	--	--	--	N/A	0.42	7.4E-04
**L5 PV**	--	--	--	--	--	--	--	--	--	N/A	0.003
**L5 SOM**	--	--	--	--	--	--	--	--	--	--	N/A

Table S4. *p* values of vM1 feedback elicited log2 normalized EPSP comparison across cell classes in vS1. Two-sided Conover's test for comparisons among cell classes other than the L2/3 pyramidal cells. Two-sided permutation test for comparisons between L2/3 pyramidal cells and other cell classes. P values were adjusted with Benjamin-Hochberg methods for multiple

comparisons.

Raw values of EPSC and EPSP show that vM1 to vS1 feedback pathway might have an overall weaker connection than LM to V1 pathway

In the main text (Fig 3), we have normalized the EPSC and EPSP amplitudes to those of L2/3 pyramidal cells to account for the variability in the amount of virus injection across the animals. In Fig S7, we show the raw values of EPSC and EPSP. Under the assumption that the strength and amount of virus injected were similar among animals, we found that EPSC and EPSP of most of the cell classes in the vM1 to vS1 pathway were significantly lower than those in LM to V1 (Fig S7, Table S5), suggesting an overall weaker connection of vM1 to vS1 feedback pathway. Note that the raw EPSC values in VIP+ cells were similar between V1 and vS1, but raw EPSP values were higher in vS1 than in V1 (p = 0.004, Mann Whitney U test, p values adjusted with Benjamin-Hochberg method). This is because the input resistance of VIP+ cells in vS1 (median 287 MΩ) is higher than that of VIP+ cells in V1 (median 204 MΩ, Fig S8, $p = 1.8\times10^{-5}$, Mann Whitney U test). With a similar amount of synaptic current, VIP+ cells in vS1 responded with a higher EPSP.

	Normalized EPSC	Normalized EPSP	Raw EPSC	Raw EPSP
L1	0.93	0.41	0.023	0.085
L2/3 Pyr	N/A	N/A	2.3E-5	0.20
L2/3 PV	0.23	0.32	0.0003	0.0087
L2/3 SOM	0.0009	0.019	0.0003	0.0087
L2/3 VIP	0.0023	0.0066	0.056	0.004
L4 Exc	0.0004	0.038	0.0003	0.038
L4 PV	0.098	0.088	0.0003	0.0057
L4 SOM	0.0004	0.0066	0.0003	0.0040
L5 Pyr	0.0004	0.041	0.043	0.66
L5 PV	0.93	0.58	0.0065	0.17
L5 SOM	0.0004	0.0066	0.0003	0.0048

Tables S5. p values of comparison of normalized and absolute EPSCs and EPSPs of the same cell type across the two feedback pathways. Two-sided permutation test, p values adjusted with Benjamin-Hochberg method.

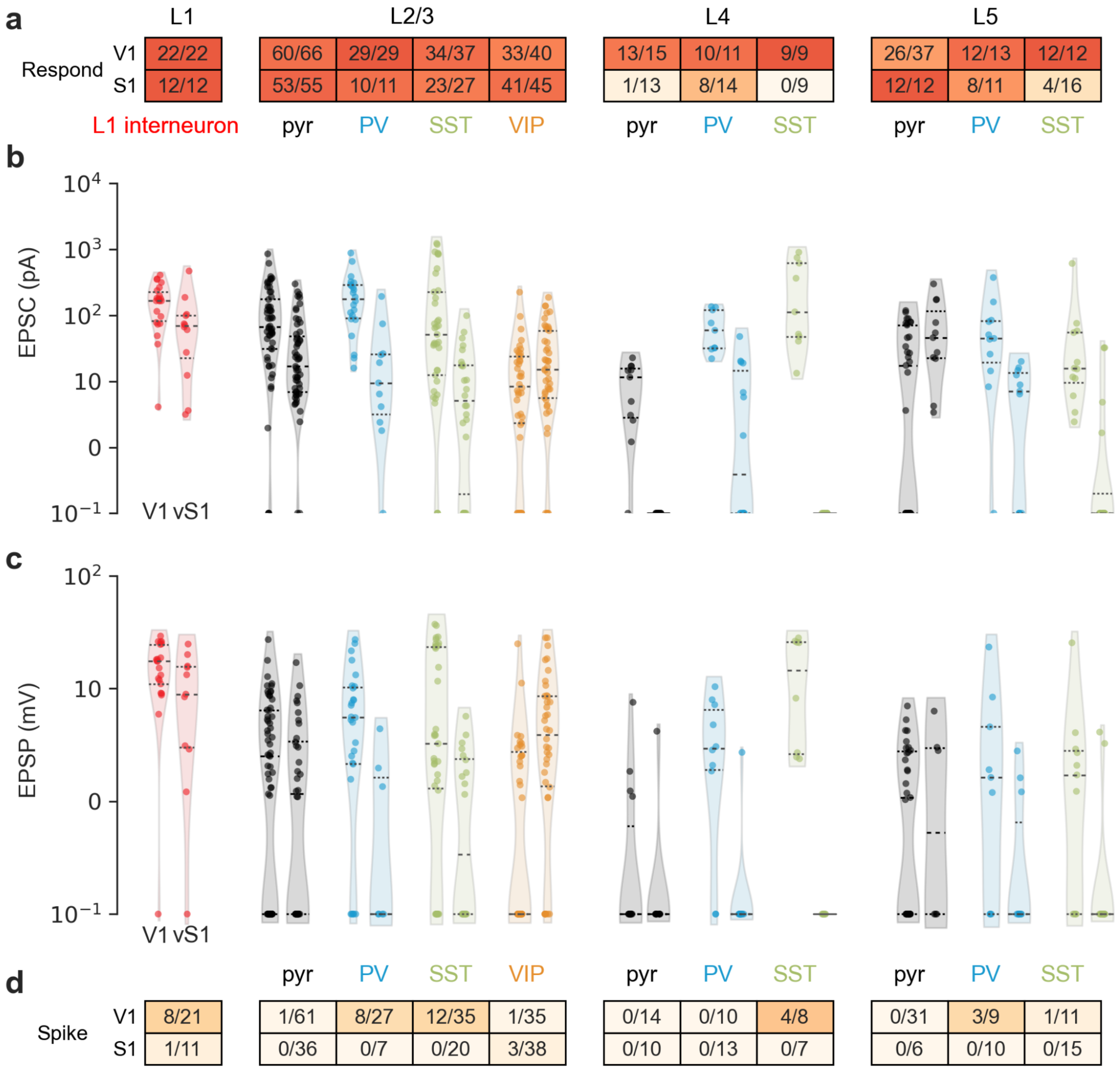

Fig S7. Summary of raw activities in V1 and vS1 in response to feedback excitation. **a** The proportion of responsive cells in V1 or vS1 in different layers (number of responsive cells/total number of recorded cells). The color in the table indicates the probability level, same for panel d. **b** Raw EPSC of cells in either V1 (left) or vS1 (right). Colors and positions of the violin plot indicate the cell type corresponding to the charts in Panels a and d. Each dot indicates one recorded cell. The black dashed lines indicate the quartiles (top and bottom) and the median (middle). The outlines indicate the distributions of raw EPSC, truncated with the observed range. **c** Same as b, for raw EPSP. **d** The proportion of spiking cells in V1 or vS1 in different layers (number of spiking cells/total cells recorded in the current-clamp mode). Source data are provided as a Source Data file.

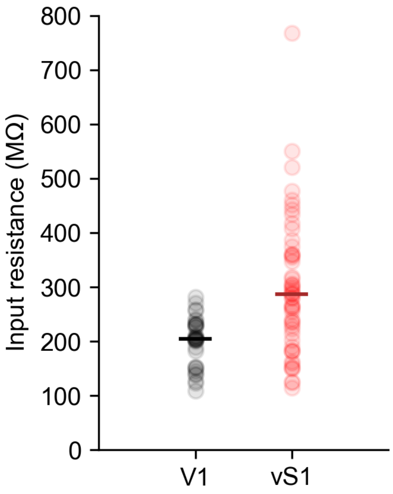

Fig S8. Input resistance of VIP+ cells in V1 or vS1. Each dot represents a cell and the bar marks the median of the population. VIP+ cells in vS1 had significantly higher input resistance than those in V1 (p = 1.8E-05, two-sided Wilcoxon rank-sum test. 34 V1 cells from 4 animals, 53 vS1 cells from 6 animals). Source data are provided as a Source Data file.

Variability of "temporal sharpening" effect across cells

In Figs S9 and S10, we show more examples of the "temporal sharpening effect". While the temporal sharpening of L2/3 and L5 pyramidal cells was highly consistent across cells (Fig S9 and Fig S10, a and c), the effect on excitatory cells in L4 was variable (Fig S9b and Fig S10b).

a

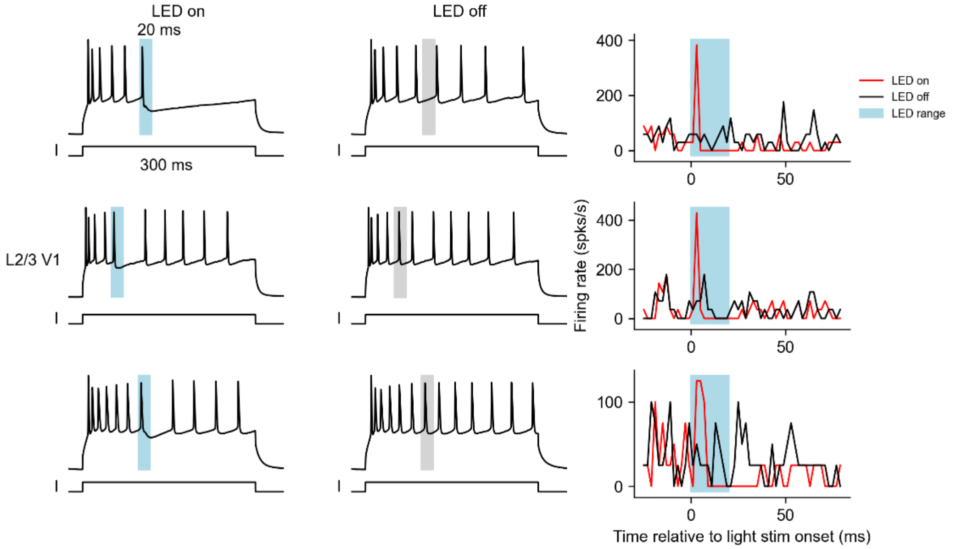
LED on
20 ms
LED off
300 ms
L2/3 V1
LED on
LED off
LED range
Firing rate (spks/s)
400
200
0
100
0
50
Time relative to light stim onset (ms)

b

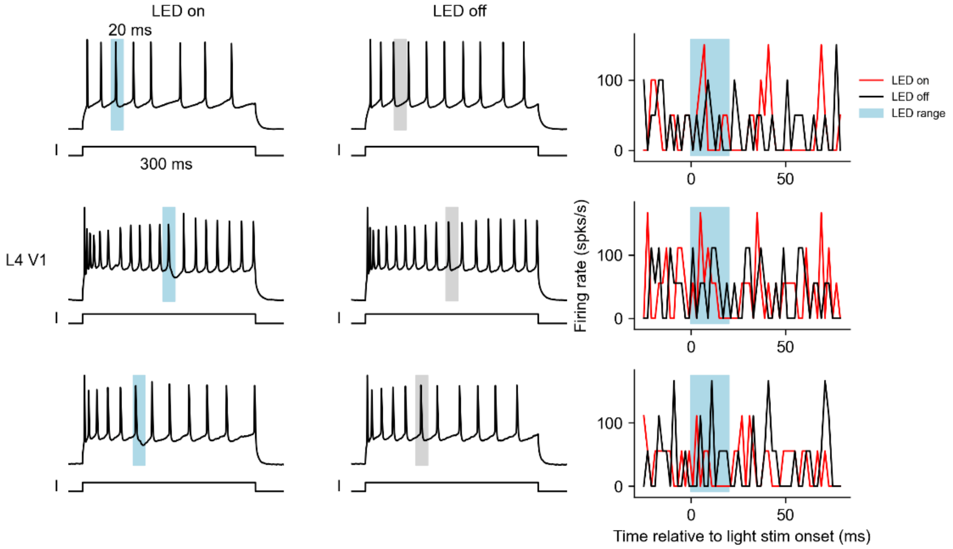
LED on
20 ms
LED off
300 ms
L4 V1
LED on
LED off
LED range
Firing rate (spks/s)
100
0
0
50
Time relative to light stim onset (ms)

c

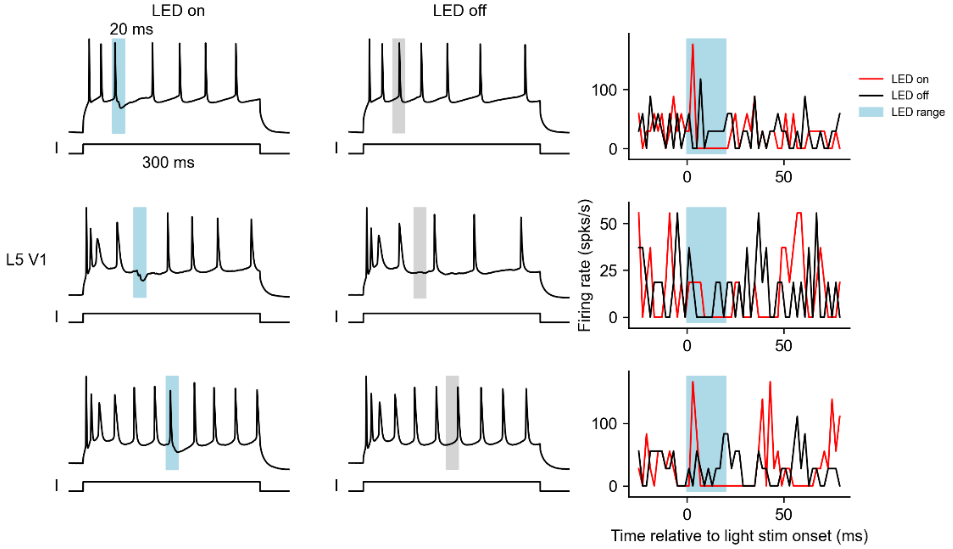
LED on
20 ms
LED off
300 ms
L5 V1
LED on
LED off
LED range
Firing rate (spks/s)
100
50
25
0
0
50
Time relative to light stim onset (ms)

Fig S9. More example V1 principal cells in L2/3 (a), L4 (b), or L5 (c) with sustained feed-forward current and brief feedback inputs. Left and Middle: example traces with LED on (left) or LED off (middle). Source data are provided as a Source Data file.

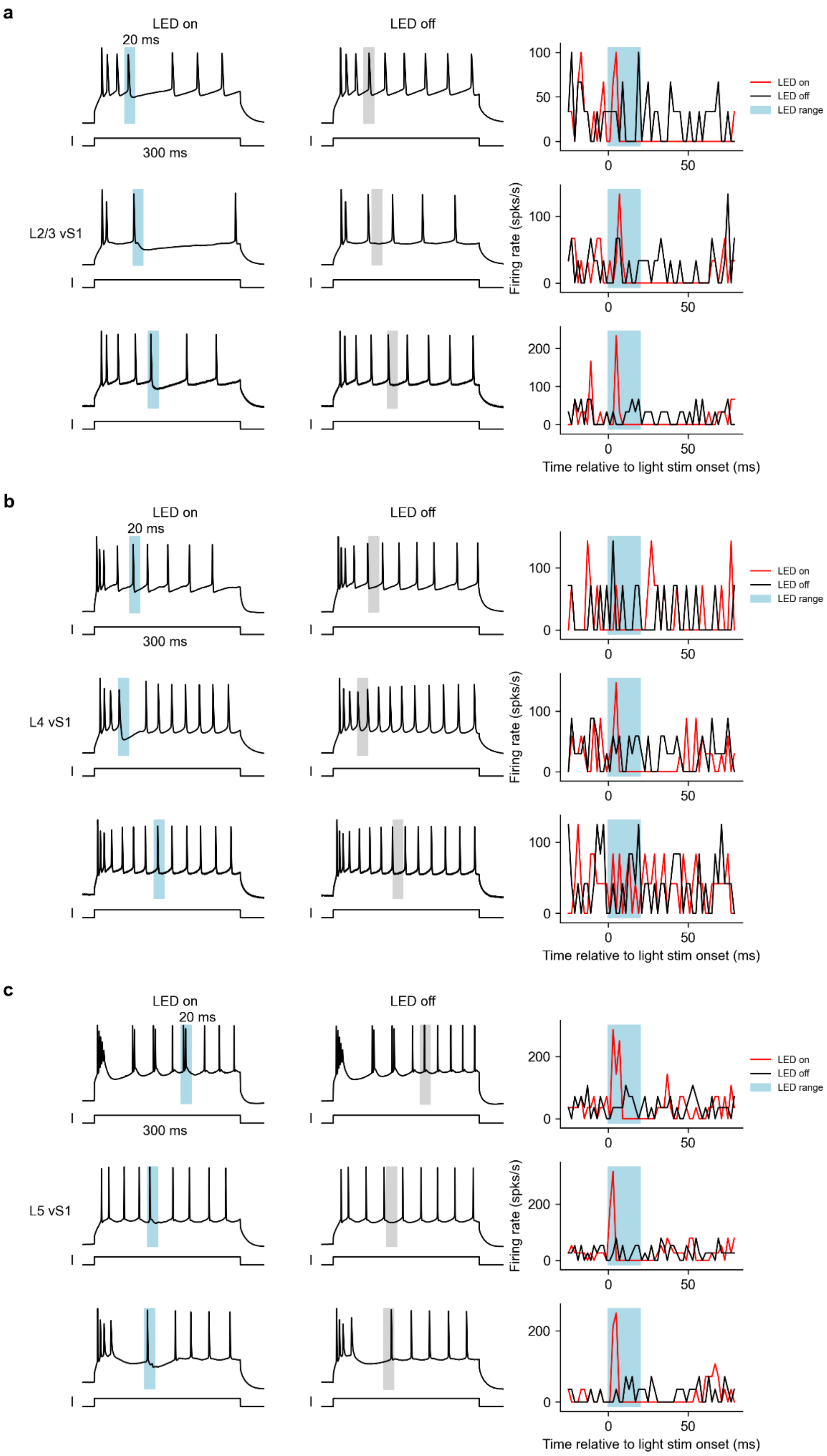
a
LED on
20 ms
LED off
300 ms
L2/3 vS1
LED on
LED off
LED range
Firing rate (spks/s)
Time relative to light stim onset (ms)
b
LED on
20 ms
LED off
300 ms
L4 vS1
Firing rate (spks/s)
Time relative to light stim onset (ms)
c
LED on
20 ms
LED off
300 ms
L5 vS1
Firing rate (spks/s)
Time relative to light stim onset (ms)

Fig S10. More example vS1 principle cells in L2/3 (a), L4 (b), or L5 (c) with sustained feed-forward current and brief feedback inputs. Left and Middle: example traces with LED on (left) or LED off (middle). Source data are provided as a Source Data file.

Comparison of burstiness for L5 IB neurons in V1 and vS1

A previous study showed differences in dendritic integration properties of L5 cells along the rostral-caudal axis in the cortex, and neurons in S1 are likely to be more prone to bursting than those cells in V1 [9]. To account for the difference in the population of L5 pyramidal cells, we preselected bursty neurons to perform the feedback stimulation protocols. These neurons, as reported in the literature, are thick-tufted neurons in both V1 [10] and vS1 [11]. To directly compare their burstiness in our experiments, we recorded the firing patterns of the neurons and measured how prone they were to burst with two metrics: minimal current to elicit a burst and burstiness. Burstiness was defined as the firing rate difference of the spikes inside bursts from that out of the bursts, normalized by their summation (see Methods). We found that there were no significant differences between V1 bursty neurons and vS1 bursty neurons in both metrics (Fig S11), indicating that the difference we found in bursting with feedback stimulation is not a result of the difference in their intrinsic bursting properties.

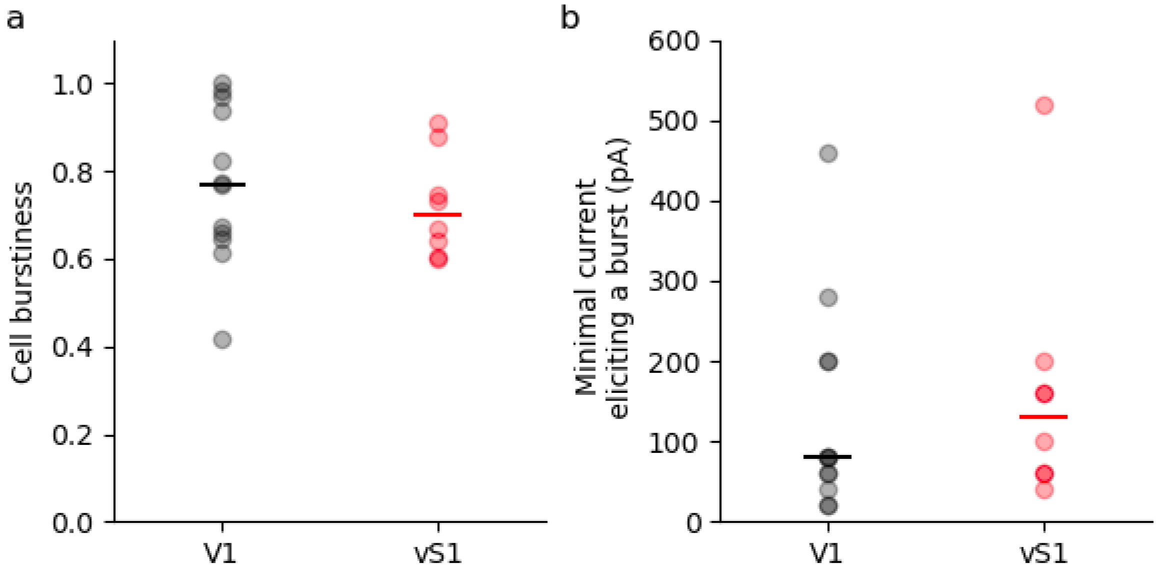

Fig S11. Characterization of intrinsic burstiness of L5 IB neurons in V1 and vS1. **a** Cell burstiness for L5 IB neurons in V1 (black) and vS1 (red). Bars mark the median values. p = 0.28, two-sided Wilcoxon rank-sum test. **b** Minimal current eliciting bursts for L5 IB neurons in V1 (black) and vS1 (red). p = 0.29, two-sided Wilcoxon rank-sum test. V1: 12 cells from 2 animals. vS1: 8 cells from 3 animals. Source data are provided as a Source Data file.

Comparison of light-evoked EPSCs of V1 and vS1 neurons in the bursting experiments

In the bursting experiments, to make the results comparable across experiments, we recorded the light-evoked EPSCs before the bursting protocol and adjusted the LED light intensity to elicit EPSC ranging from 100 pA to 500 pA. We showed in Fig S12 that there was no significant difference in EPSC between cells in V1 and vS1 (Fig S12).

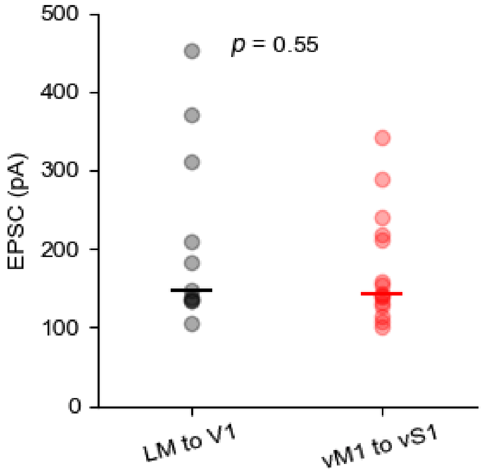

Fig S12. Light-evoked EPSCs of V1 and vS1 intrinsic bursty neurons. The bars mark the median values. p = 0.55, two-sided Wilcoxon rank-sum test. V1: 11 cells from 2 animals. S1: 15 cells from 5 animals. Source data are provided as a Source Data file.

Rise time, decay time and time to peak

To identify whether the difference we found in feedback-evoked bursting was because of the difference in the excitatory response of L5 cells, we measured the rise time, decay time and time to peak for feedback eliciting EPSC in L5 pyramidal cells, but found no significant difference in those measures (Fig S13). This result indicates the difference in the bursting was more likely due to the difference in feedback-evoked inhibitory circuits.

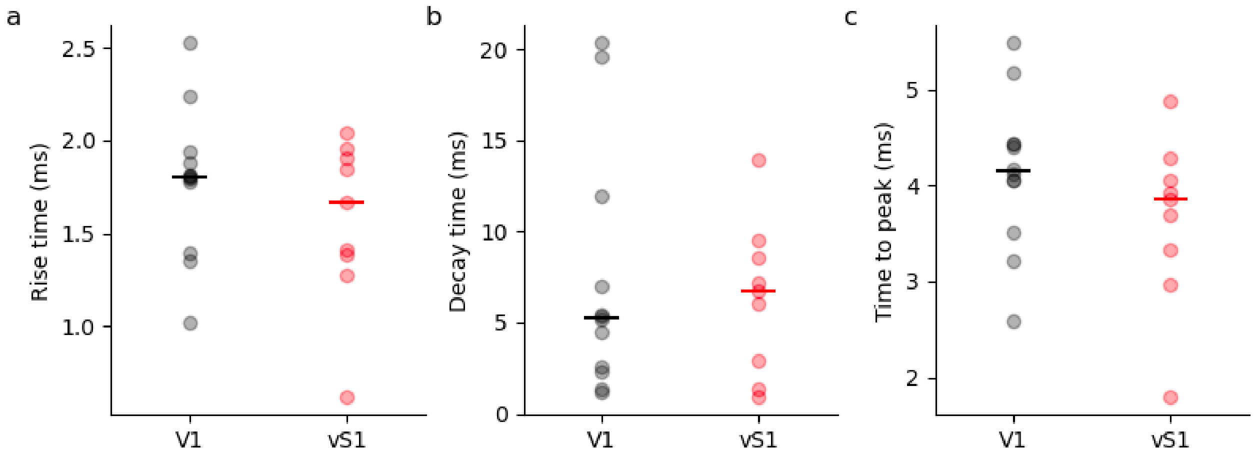

Fig S13. Rise time **(a),** decay time **(b)** and time to peak **(c)** for L5 pyramidal cells in V1 (black) and vS1 (red). V1: 12 cells from 5 animals. vS1: 9 cells from 3 animals. Rise time comparison in (a): p = 0.57, two-sided Wilcoxon rank-sum test. Decay time comparison in (v): p = 0.72, two-sided Wilcoxon rank-sum test. Time to peak comparison in (c): p = 0.12, two-sided Wilcoxon rank-sum test.

Supplementary References

1. Jiang, X., Shen, S., Cadwell, C. R., Berens, P. & Sinz, F. Principles of connectivity among morphologically defined cell types in adult neocortex. (2015).
2. Reimer, J. *et al.* Pupil Fluctuations Track Fast Switching of Cortical States during Quiet Wakefulness. *Neuron* **84**, 355–362 (2014).
3. Large, A. M., Kunz, N. A., Mielo, S. L. & Oswald, A.-M. M. Inhibition by Somatostatin Interneurons in Olfactory Cortex. *Front. Neural Circuits* **10**, 62 (2016).
4. Petreanu, L., Mao, T., Sternson, S. M. & Svoboda, K. The subcellular organization of neocortical excitatory connections. *Nature* vol. 457 1142–1145 (2009).
5. Cho, J.-H., Deisseroth, K. & Bolshakov, V. Y. Synaptic encoding of fear extinction in mPFC-amygdala circuits. *Neuron* **80**, 1491–1507 (2013).
6. Lee, S., Kruglikov, I., Huang, Z. J., Fishell, G. & Rudy, B. A disinhibitory circuit mediates

motor integration in the somatosensory cortex. *Nat. Neurosci.* **16**, 1662–1670 (2013).

7. Ballanyi, K. & Ruangkittisakul, A. Brain Slices. in *Encyclopedia of Neuroscience* (eds. Binder, M. D., Hirokawa, N. & Windhorst, U.) 483–490 (Springer Berlin Heidelberg, 2009).

8. Ting, J. T., Daigle, T. L., Chen, Q. & Feng, G. Acute Brain Slice Methods for Adult and Aging Animals: Application of Targeted Patch Clamp Analysis and Optogenetics. *Patch-Clamp Methods and Protocols* **1183**, 221–242 (2014).

9. Fletcher, L. N. & Williams, S. R. Neocortical Topology Governs the Dendritic Integrative Capacity of Layer 5 Pyramidal Neurons. *Neuron* **101**, 76–90.e4 (2019).

10. Shai, A. S., Anastassiou, C. A., Larkum, M. E. & Koch, C. Physiology of layer 5 pyramidal neurons in mouse primary visual cortex: coincidence detection through bursting. *PLoS Comput. Biol.* **11**, e1004090 (2015).

11. Oberlaender, M. *et al.* Three-dimensional axon morphologies of individual layer 5 neurons indicate cell type-specific intracortical pathways for whisker motion and touch. *Proc. Natl. Acad. Sci. U. S. A.* **108**, 4188–4193 (2011).
