# Supplementary material for: Distinct organization of two cortico-cortical feedback pathways
Source: Nat Commun. 2022 Oct 27;13:6389. doi: 10.1038/s41467-022-33883-9 (PMC9613627; doi:10.1038/s41467-022-33883-9)
Supplement: Supplementary file 3 — Reporting Summary [file 41467_2022_33883_MOESM3_ESM.pdf]

nature portfolio

Corresponding author(s): Andreas Tolias

Last updated by author(s): 09/22/2022

Reporting Summary

Nature Portfolio wishes to improve the reproducibility of the work that we publish. This form provides structure for consistency and transparency in reporting. For further information on Nature Portfolio policies, see our Editorial Policies and the Editorial Policy Checklist.

Statistics

For all statistical analyses, confirm that the following items are present in the figure legend, table legend, main text, or Methods section.

n/a	Confirmed	
☐	☒	The exact sample size (n) for each experimental group/condition, given as a discrete number and unit of measurement
☐	☒	A statement on whether measurements were taken from distinct samples or whether the same sample was measured repeatedly
☐	☒	The statistical test(s) used AND whether they are one- or two-sided<br>*Only common tests should be described solely by name; describe more complex techniques in the Methods section.*
☐	☒	A description of all covariates tested
☐	☒	A description of any assumptions or corrections, such as tests of normality and adjustment for multiple comparisons
☐	☒	A full description of the statistical parameters including central tendency (e.g. means) or other basic estimates (e.g. regression coefficient) AND variation (e.g. standard deviation) or associated estimates of uncertainty (e.g. confidence intervals)
☐	☒	For null hypothesis testing, the test statistic (e.g. F, t, r) with confidence intervals, effect sizes, degrees of freedom and P value noted<br>*Give P values as exact values whenever suitable.*
☒	☐	For Bayesian analysis, information on the choice of priors and Markov chain Monte Carlo settings
☒	☐	For hierarchical and complex designs, identification of the appropriate level for tests and full reporting of outcomes
☒	☐	Estimates of effect sizes (e.g. Cohen's d, Pearson's r), indicating how they were calculated

Our web collection on statistics for biologists contains articles on many of the points above.

Software and code

Policy information about availability of computer code

Data collection	We used Psychtoolbox in MATLAB (R2022a) to generate grating stimuli. The code is available in the GitHub repository https://github.com/shenshan/visual_stimuli
Data analysis	We published the processed data and code and to replicate the essential figures in the GitHub Repository https://github.com/shenshan/feedback_paper_codeshare, based on Python 3.8.8<br>To analyze the burstiness of V1 and vS1 excitatory cells, we referenced Allen SDK, hosted with repository https://github.com/alleninstitute/allensdk, run on Python 3.8.8

For manuscripts utilizing custom algorithms or software that are central to the research but not yet described in published literature, software must be made available to editors and reviewers. We strongly encourage code deposition in a community repository (e.g. GitHub). See the Nature Portfolio guidelines for submitting code & software for further information.

Data

Policy information about availability of data

All manuscripts must include a data availability statement. This statement should provide the following information, where applicable:

- Accession codes, unique identifiers, or web links for publicly available datasets
- A description of any restrictions on data availability
- For clinical datasets or third party data, please ensure that the statement adheres to our policy

We published the processed data and code and to replicate the essential figures in the GitHub Repository https://github.com/shenshan/feedback_paper_codeshare
The full data processing pipeline is available from the corresponding author on reasonable request.

Field-specific reporting

Please select the one below that is the best fit for your research. If you are not sure, read the appropriate sections before making your selection.

- [x] Life sciences
- [ ] Behavioural & social sciences
- [ ] Ecological, evolutionary & environmental sciences

For a reference copy of the document with all sections, see nature.com/documents/nr-reporting-summary-flat.pdf

Life sciences study design

All studies must disclose on these points even when the disclosure is negative.

Sample size	We performed power analyses to estimate the sample size we used, with consideration of statistical power, significance level, minimal meaningful effect size and variability of data.
Data exclusions	For all experiments, we excluded animals with poor injection quality, including low expression level or virus overspilling to areas outside the target.
Replication	In each of the experiments, we performed replications by recording from multiple cells and multiple animals to ensure reproducibility. The effects we reported in this study were reproducible across neurons and animals. We listed the number of independent experiments we performed for each experiment in the figure legends.
Randomization	We used the same species and strain of mice within a certain age range throughout the study. For each experimental group, the sex and age of the animals where randomized to reduce the effects from the covariates.
Blinding	Blinding is not possible for our study because we have to intentionally record from different brain areas and cell types, but this should not affect our results because we performed exactly the same protocol on different experimental groups.

Reporting for specific materials, systems and methods

We require information from authors about some types of materials, experimental systems and methods used in many studies. Here, indicate whether each material, system or method listed is relevant to your study. If you are not sure if a list item applies to your research, read the appropriate section before selecting a response.

Materials & experimental systems

n/a	Involved in the study	
[x]	[ ]	Antibodies
[x]	[ ]	Eukaryotic cell lines
[x]	[ ]	Palaeontology and archaeology
[ ]	[x]	Animals and other organisms
[x]	[ ]	Human research participants
[x]	[ ]	Clinical data
[x]	[ ]	Dual use research of concern

Methods

n/a	Involved in the study	
[x]	[ ]	ChIP-seq
[x]	[ ]	Flow cytometry
[x]	[ ]	MRI-based neuroimaging

Animals and other organisms

Policy information about studies involving animals; ARRIVE guidelines recommended for reporting animal research

Laboratory animals	In this study, we used 79 mice (Mus musculus) in total (51 males and 28 females), aged 8 weeks to 4 months. These included 4 C57Bl/6 mice (all male), 29 PV-Cre/Ai9 mice (19 males and 10 females), 35 SOM-Cre/Ai9 mice (22 males and 13 females), and 11 VIP-Cre/Ai9 mice (6 males and 5 females). All Cre and Ai9 reporter lines are on a C57Bl/6 background. The animals were bred and maintained in the facility with a light cycle of 6am to 6pm daily, a temperature ranging from 68 to 72 Fahrenheit and a humidity ranging from 30% to 70%.
Wild animals	The study did not involve wild animals.
Field-collected samples	The study did not involve field-collected samples.
Ethics oversight	All procedures performed on animals were in accordance with the ethical guidelines of the National Institutes of Health and were approved by the Institutional Animal Care and Use Committee (IACUC) of Baylor College of Medicine.

Note that full information on the approval of the study protocol must also be provided in the manuscript.
