# Supplementary figures and images for: Distinct organization of two cortico-cortical feedback pathways
Source: Nat Commun. 2022 Oct 27;13:6389. doi: 10.1038/s41467-022-33883-9 (PMC9613627; doi:10.1038/s41467-022-33883-9)

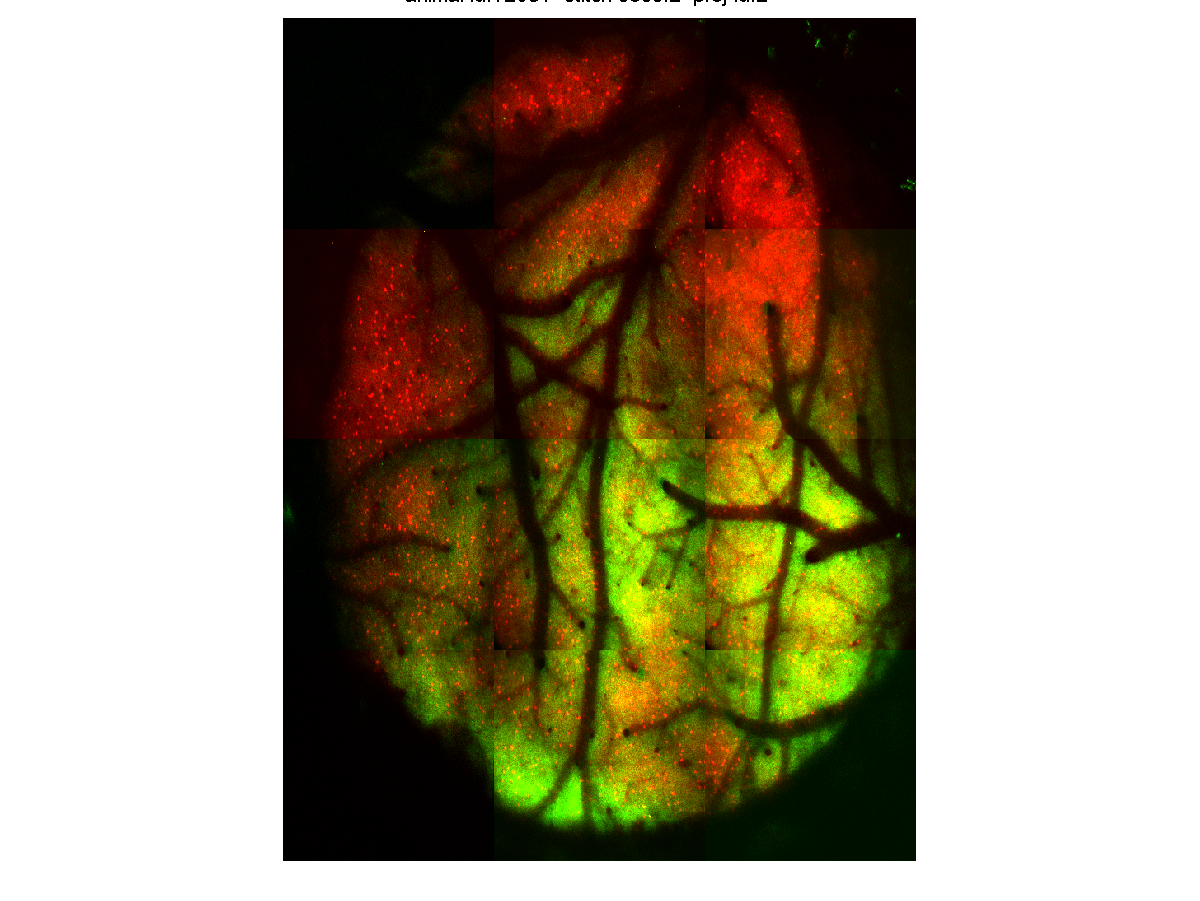

Supplement: Supplementary file 4 — Source Data [file 41467_2022_33883_MOESM4_ESM.zip › Source_Data_File/figure_1d_12051_infection.png]

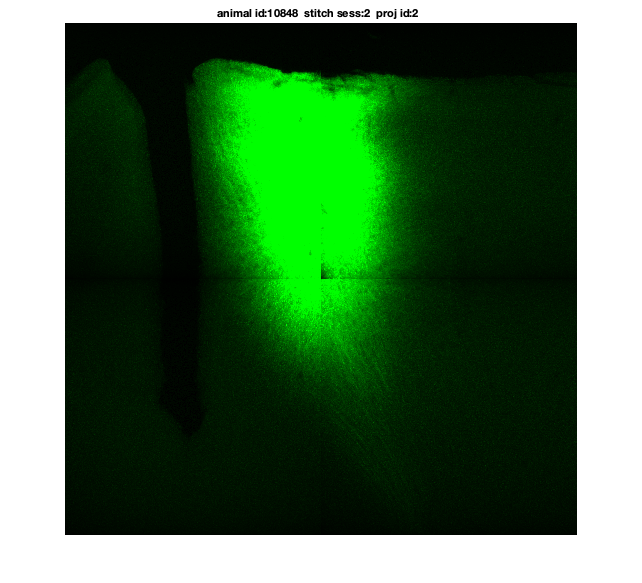

Supplement: Supplementary file 4 — Source Data [file 41467_2022_33883_MOESM4_ESM.zip › Source_Data_File/figure_s1b_right_10848_vM1_infection.png]

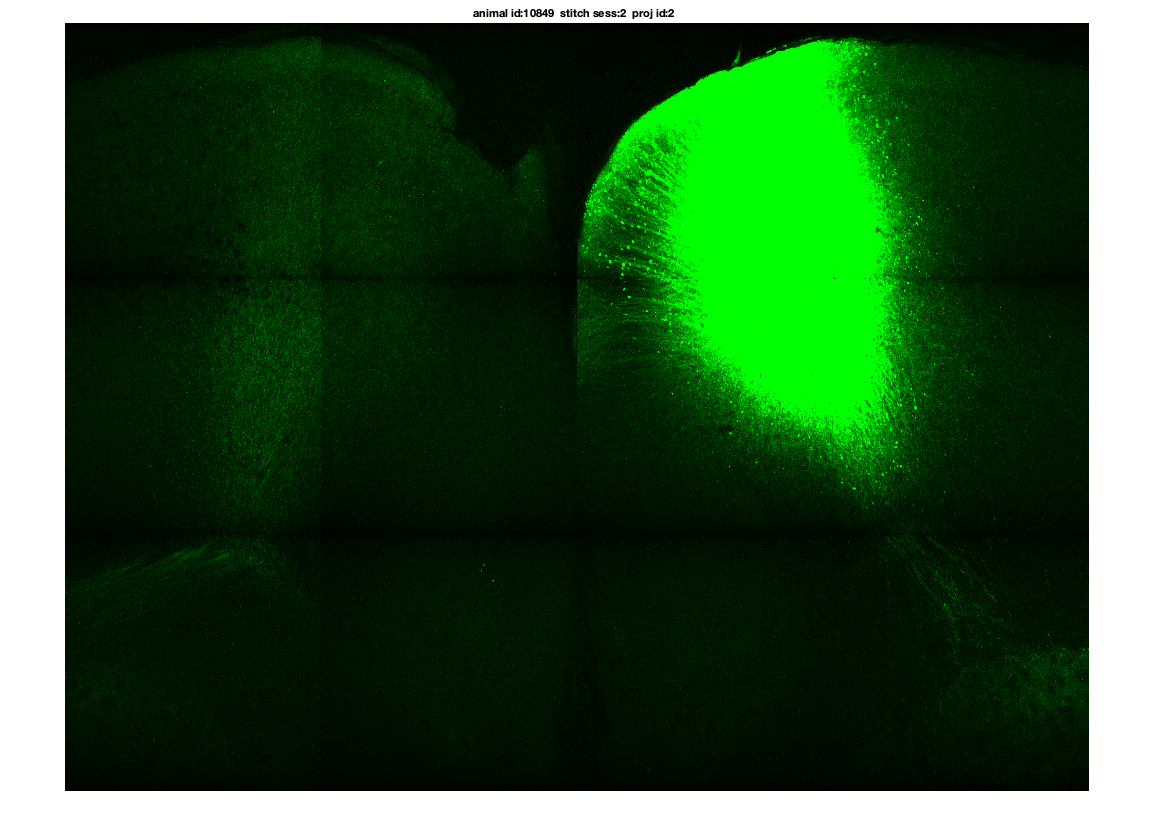

Supplement: Supplementary file 4 — Source Data [file 41467_2022_33883_MOESM4_ESM.zip › Source_Data_File/figure_s1b_left_10849_vM1_injection.png]

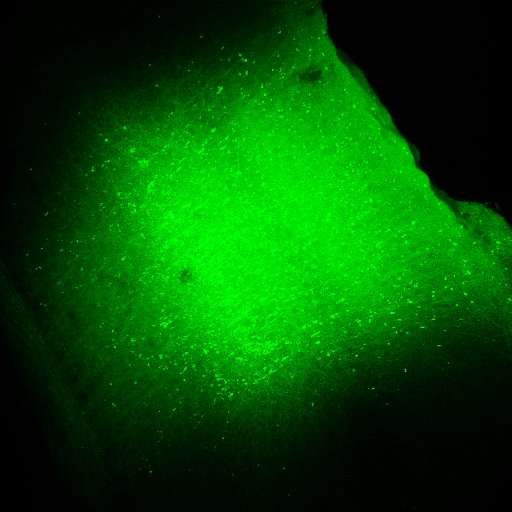

Supplement: Supplementary file 4 — Source Data [file 41467_2022_33883_MOESM4_ESM.zip › Source_Data_File/figure_s1a_right_5213_infection_lm.jpg]

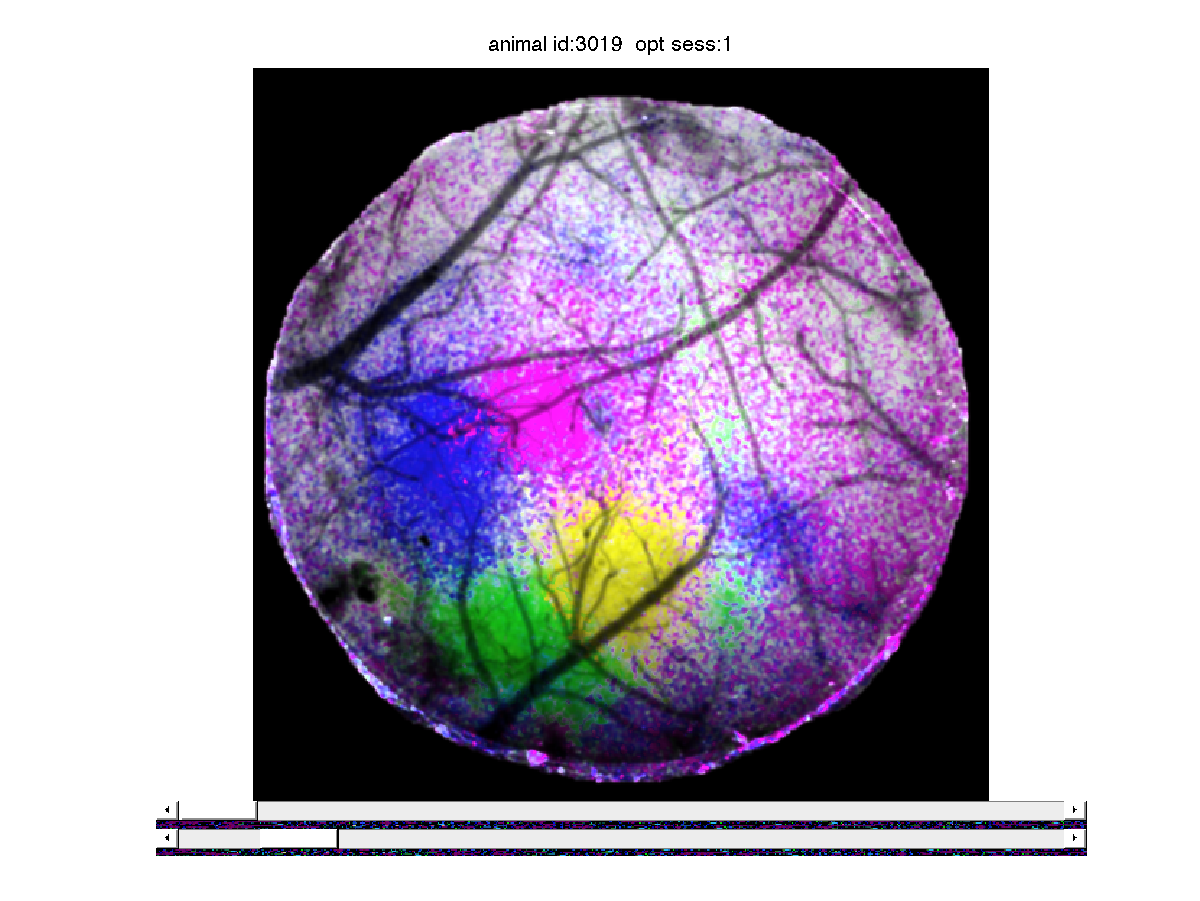

Supplement: Supplementary file 4 — Source Data [file 41467_2022_33883_MOESM4_ESM.zip › Source_Data_File/figure_1b_3019_intrinsic.png]

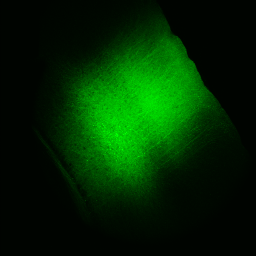

Supplement: Supplementary file 4 — Source Data [file 41467_2022_33883_MOESM4_ESM.zip › Source_Data_File/figure_s1a_left_1627_infection_lm.png]

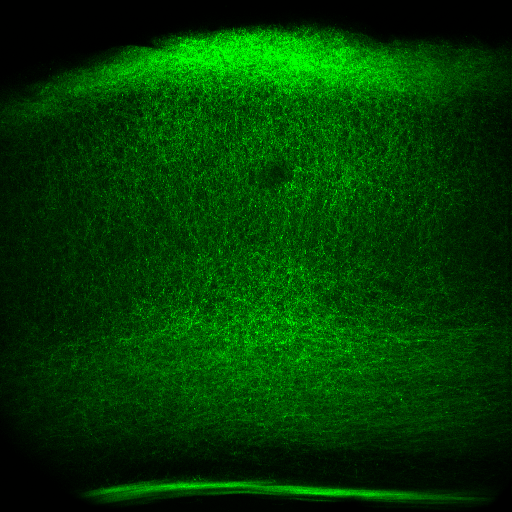

Supplement: Supplementary file 4 — Source Data [file 41467_2022_33883_MOESM4_ESM.zip › Source_Data_File/figure_1e_left_5213_slice.png]
